# Changes in mRNA expression of arcuate nucleus appetite-regulating peptides during lactation in rats

**DOI:** 10.1530/JME-13-0015

**Published:** 2014-04

**Authors:** Yoshihiro Suzuki, Keiko Nakahara, Keisuke Maruyama, Rieko Okame, Takuya Ensho, Yoshiyuki Inoue, Noboru Murakami

**Affiliations:** 1 Department of Veterinary Physiology, Faculty of AgricultureUniversity of MiyazakiMiyazaki, 889-2192Japan

**Keywords:** lactation, hypothalamus, arcuate nucleus, AgRP, POMC, C3, PYY, leptin

## Abstract

The contribution of hypothalamic appetite-regulating peptides to further hyperphagia accompanying the course of lactation in rats was investigated by using PCR array and real-time PCR. Furthermore, changes in the mRNA expression for appetite-regulating peptides in the hypothalamic arcuate nucleus (ARC) were analyzed at all stages of pregnancy and lactation, and also after weaning. Food intake was significantly higher during pregnancy, lactation, and after weaning than during non-lactation periods. During lactation, ARC expression of mRNAs for agouti-related protein (AgRP) and peptide YY was increased, whereas that of mRNAs for proopiomelanocortin (POMC) and cholecystokinin (CCK) was decreased, in comparison with non-lactation periods. The increase in AgRP mRNA expression during lactation was especially marked. The plasma level of leptin was significantly decreased during the course of lactation, whereas that of acyl-ghrelin was unchanged. In addition, food intake was negatively correlated with the plasma leptin level during lactation. This study has clarified synchronous changes in the expression of many appetite-regulating peptides in ARC of rats during lactation. Our results suggest that hyperphagia during lactation in rats is caused by decreases in POMC and CCK expression and increases in AgRP expression in ARC, the latter being most notable. Together with the decrease in the blood leptin level, such changes in mRNA expression may explain the further hyperphagia accompanying the course of lactation.

## Introduction

In lactating mammals, many physiological changes occur in order to ensure milk production for suckling. These include development of the mammary glands, and increases in the plasma levels of prolactin, the hormone that triggers milk production, and oxytocin, the hormone that triggers milk ejection, in response to the suckling stimulus (Higuchi *et al*. 1985, Lee *et al*. 1989). Growth hormone (GH) also plays an important role in milk production (Flint *et al*. 1992, Etherton & Bauman 1998). Lactation in the mammary gland also markedly increases the nutrient requirement of female mammals to a level that exceeds that of the whole body in non-lactating females (Vernon & Flint 1984, Wade & Schneider 1992). As a consequence, food consumption increases several fold during lactation (Asakuma *et al*. 2004, Woodside *et al*. 2012). Furthermore, daily food consumption increases with milk yield and pup growth (Woodside 2007). Although rats feed mainly at night, lactating females eat as much in the daytime as they do at night (Munday & Williamson 1983). In order to induce this change in feeding behavior, various alterations occur in the CNS.

The hypothalamus has an important role in appetite regulation. For example, damage to the ventromedial hypothalamus causes hyperphagia, whereas destruction of the lateral hypothalamus results in profound anorexia (Hetherington & Ranson 1940, Anand & Brobeck 1951). The control of appetite by the hypothalamus involves many peptides. Increases in the protein levels of neuropeptide Y (NPY) and agouti-related protein (AgRP) trigger an orexigenic response, whereas increases in the levels of proopiomelanocortin (POMC) and cocaine- and amphetamine-regulated transcript trigger an anorexigenic response (Ellacott & Cone 2004). Especially, the hypothalamic arcuate nucleus (ARC), one of the crucial sites in the hypothalamus regulating appetite and body weight, contains both NPY/AgRP and POMC neurons. In addition, the neurons in the ARC send axons to the paraventricular nucleus, dorsomedial nucleus, and lateral hypothalamic area (Kawano *et al*. 2002, Ellacott & Cone 2004). The expression of appetite-related genes is regulated by a number of endocrine substances, which including leptin, acyl-ghrelin, complement C3 (C3), cholecystokinin (CCK), galanin, hypocretin, and peptide YY (PYY) (Smith & Ferguson 2008, Ricklin *et al*. 2010, Parker & Bloom 2012).

Leptin is an anorexigenic hormone primarily secreted from white adipose tissue (WAT). In addition, synthesis of leptin occurs in brown adipose tissue, liver, skeletal muscle, the gastric fundus, placenta, ovary, mammary epithelial cells, bone marrow, pituitary gland, and hypothalamus (Morash *et al*. 1999). The effects of leptin are mediated by binding to OBRb (Tartaglia *et al*. 1995, Lee *et al*. 1996). Leptin is implicated in the regulation of energy metabolism and energy expenditure (Cohen *et al*. 2002, Chilliard *et al*. 2005), food intake (Pelleymounter *et al*. 1995, Levin *et al*. 1996), and reproduction (Clarke & Henry 1999). These effects are exerted via the CNS and in order for this to occur peripheral leptin must be transported across the blood–brain barrier (BBB) and/or arrive in the solitary tract nucleus (NTS) via the vagal afferent pathway (Buyse *et al*. 2001). For hypophagia to occur, leptin increases the hypothalamic expression of POMC mRNA and decreases that of NPY and AgRP mRNAs (Schwartz *et al*. 1996, 1997, Thornton *et al*. 1997).

In lactating female rats, it has been reported that peripheral prolactin, PYY, and CCK levels are increased compared with those in non-lactating female (Lee *et al*. 1989, Lindén *et al*. 1990, Taylor *et al*. 2009). Many studies have demonstrated that lactation in rats does not change, nor reduce, acyl-ghrelin levels in blood (Abizaid *et al*. 2008, Taylor *et al*. 2009). The blood leptin level in lactating rats was shown to be lower than that in non-lactating females (Pickavance *et al*. 1998). Furthermore, our previous study demonstrated that i.v. administration of leptin to lactating rats did not suppress food intake, suggesting that the hyperphagia shown by lactating rats may be related to a decline in the anorexigenic effect of leptin (Suzuki *et al*. 2010). These observations raise the possibility that these changes in peripheral hormones levels are involved in central feeding control on the hyperphagia during lactation.

Many researchers have investigated the effects of leptin, ghrelin, prolactin, CCK, and sucking stimulation on hypothalamic appetite-regulating peptides during lactation (Chen *et al*. 2008, Crowley *et al*. 2007, Woodside 2007, Woodside *et al.* 2012, Cui *et al*. 2011). However, the factors directly mediating the continued hyperphagia accompanying the course of lactation have not been fully elucidated. The aim of this study, therefore, was to clarify this issue through a global analysis of mRNA expression for hypothalamic appetite-regulating peptides and of peripheral hormone levels at two different phases of lactation: early (day 5) and late (day 15).

## Materials and methods

### Animals

All experiments were conducted in accordance with the Japanese Physiological Society's guidelines for animal care. Female Wistar rats aged 13–18 weeks were used. The rats were housed under constant temperature (23±1 °C) and a 12 h light:12 h darkness lighting regime (light on at 0700 h) with free access to food and water. The female rats were mated on the day of proestrus at the age of 10–11 weeks, and the day after mating was counted as day 1 of pregnancy. Rats on day 14 of pregnancy were used in Experiment 3. Lactating rats were mated at 9–10 weeks of age and housed singly with their litters at 13–16 weeks of age. The litter size was adjusted to ten pups (five males and five females) at birth. The day of parturition was counted as day 0 of lactation. Rats were used on day 5 or 15 of lactation. Pups were weaned on day 21 of lactation. The day of weaning was counted as day 0 after weaning. Rats of day 9 after weaning were used in Experiment 3. The control (non-lactating) rats were virgin, at the diestrus stage, and the same age as the lactating rats.

### Experiment 1: changes in mRNA expression for hypothalamic appetite-regulating peptides in lactating and non-lactating rats

The body weights and 24-h food intakes of lactating and non-lactating rats were measured at 1000 h, and the animals were then decapitated to obtain blood and brain samples. Abdominal WAT weights were also measured. Blood samples were placed in a tube containing EDTA-2Na (2 mg/ml blood) and aprotinin (500 KIU/ml blood) and centrifuged at 4 °C. To determine the plasma concentration of acyl-ghrelin, plasma was immediately mixed with a 1/10 volume of 1 M HCl. The brain samples were immediately divided into hypothalamic blocks and total RNA was extracted from each block using TRIzol (Life Technologies Co.), and then purified using an RNeasy Plus Micro Kit (Qiagen). Plasma samples were stored at −20 °C until analyzed. The samples of total RNA were stored at −80 °C until analyzed by PCR array and real-time RT-PCR.

### Experiment 2: changes in daily food intake and plasma leptin level during lactation

Daily food intakes and body weights of dams were measured at 1000 h until day 26 (day 5 after weaning). After measurement of food intake and body weight, blood samples were collected from dams daily in heparinized capillary tubes by the tail tip incision method. Blood samples were centrifuged at 4 °C, and plasma samples were stored at −20 °C until analysis of plasma leptin levels.

### Experiment 3: changes in mRNA expression for appetite-regulating peptides in the ARC at all stages of pregnancy and lactation, and after weaning

Body weights and 24-h food intakes were determined daily at 1000 h for pregnant and lactating dams, and also day 9 after weaning; those for non-lactating rats were also measured. ARC samples were immediately obtained from the brain by punch-out, which takes a target part from the brain slice. Total RNA was extracted from each ARC sample using TRIzol (Life Technologies Co.), and purified using an RNeasy Plus Micro Kit (Qiagen). The samples of total RNA were stored at −80 °C until analyzed by real-time RT-PCR. The diagram of each experiment timeline was shown in Supplementary Figure 1, see section on supplementary data given at the end of this article.

### PCR array analysis

Total RNA (1 μg in a final volume of 111 μl) was reverse-transcribed into first-strand cDNA using a RT^2^ First-Strand Kit (Qiagen). PCR array was carried out using a Rat Obesity RT^2^ Profiler PCR Array (Qiagen).

### Real-time RT-PCR analysis

Real-time RT-PCR was used to quantify changes in the expression of eight genes that were chosen based on the results of PCR array analysis (Table 1). These genes, except for *Npy* gene, fulfill the following three criteria: i) genes of peptides excluding receptors, ii) ANOVA indicated significant differences in mRNA expression levels between groups, and iii) lactation changed mRNA expression levels by 25% or more.

Total RNA (2 μg in a final volume of 10 μl) was reverse-transcribed into first-strand cDNA using a High Capacity cDNA RT Kit (Applied Biosystems). Real-time quantitative PCR was carried out using TaqMan Universal Master Mix II (Applied Biosystems) with primers to amplify *Npy*, *Agrp*, galanin, hypocretin, *Pomc*, *C3*, *Cck*, *Pyy*, and lactate dehydrogenase A (*Ldha*). For these nine genes, probe/primer kits were purchased from Applied Biosystems (TaqMan Gene Expression Assay ID: Rn00561681_m1, GenBank NM: NM_012614 for Npy, Assay ID: Rn01431703_g1, GenBank NM: NM_033650 for AgRP, Assay ID: Rn0583681_m1, GenBank NM: NM_033237 for Galanin, Assay ID: Rn00565995_m1, GenBank NM: NM_013179 for hypocretin, Assay ID: Rn00595020_m1, GenBank NM: NM_139326 for POMC, Assay ID: Rn0566466_m1, GenBank NM: NM_016994 for C3, Assay ID: Rn0563215_m1, GenBank NM: NM_012829 for CCK, Assay ID: Rn01460420_g1, GenBank NM: NM_001034080 for PYY, and Assay ID: Rn00820751_g1, GenBank NM: NM_017025 for Ldha).

### Biochemical analysis of plasma glucose, triglyceride, and total cholesterol

Plasma glucose, triglyceride (TG), and total cholesterol were determined using a FUJIFILM DRI-CHEM3500V (Fuji Film Co., Tokyo, Japan).

### Analysis of plasma leptin, acyl-ghrelin, prolactin, *Pyy*, *Cck*, and *Gh*

Plasma leptin, acyl-ghrelin, prolactin, *Pyy*, *Cck*, and *Gh* were determined using a rat leptin ELISA Kit (Morinaga Institute of Biological Science, Inc., Kanagawa, Japan), an acyl-ghrelin ELISA Kit (Mitsubishi Chemical Medience Co., Tokyo, Japan), a rat prolactin EIA Kit (SPI-BIO, Montigny Le Bretonneux, France), a mouse/rat PYY EIA Kit (Yanaihara Institute, Inc., Shizuoka, Japan), a CCK (26–33) EIA Kit (Phoenix Pharmaceuticals, Inc., Burlingame, CA, USA), and a rat/mouse GH ELISA Kit (EMD Millipore Co., Billerica, MA, USA) following each of the manufacturer's protocols.

### Statistical analysis

All data were expressed as the mean±s.e.m. In Experiment 1, differences between groups were analyzed by one-way ANOVA, *post hoc* Fisher's test, or Tukey's test. In Experiment 2, chronological changes in body weight, food intake, and plasma leptin level during lactation were analyzed by one-way repeated ANOVA. Differences in data before and after weaning were analyzed by Dunnett's test. In the before weaning group, data for body weight and plasma leptin level before weaning were taken on day 21 of lactation, and data for food intake were taken on day 16 of lactation. The correlations between food intake and leptin level were analyzed by Pearson's correlation. In Experiment 3, differences between non-lactating and other groups were analyzed by Dunnett's test, and changes between time points during lactation (days 5 and 15) were analyzed by *t*-test.

## Results

### Experiment 1

**Figure 1:**
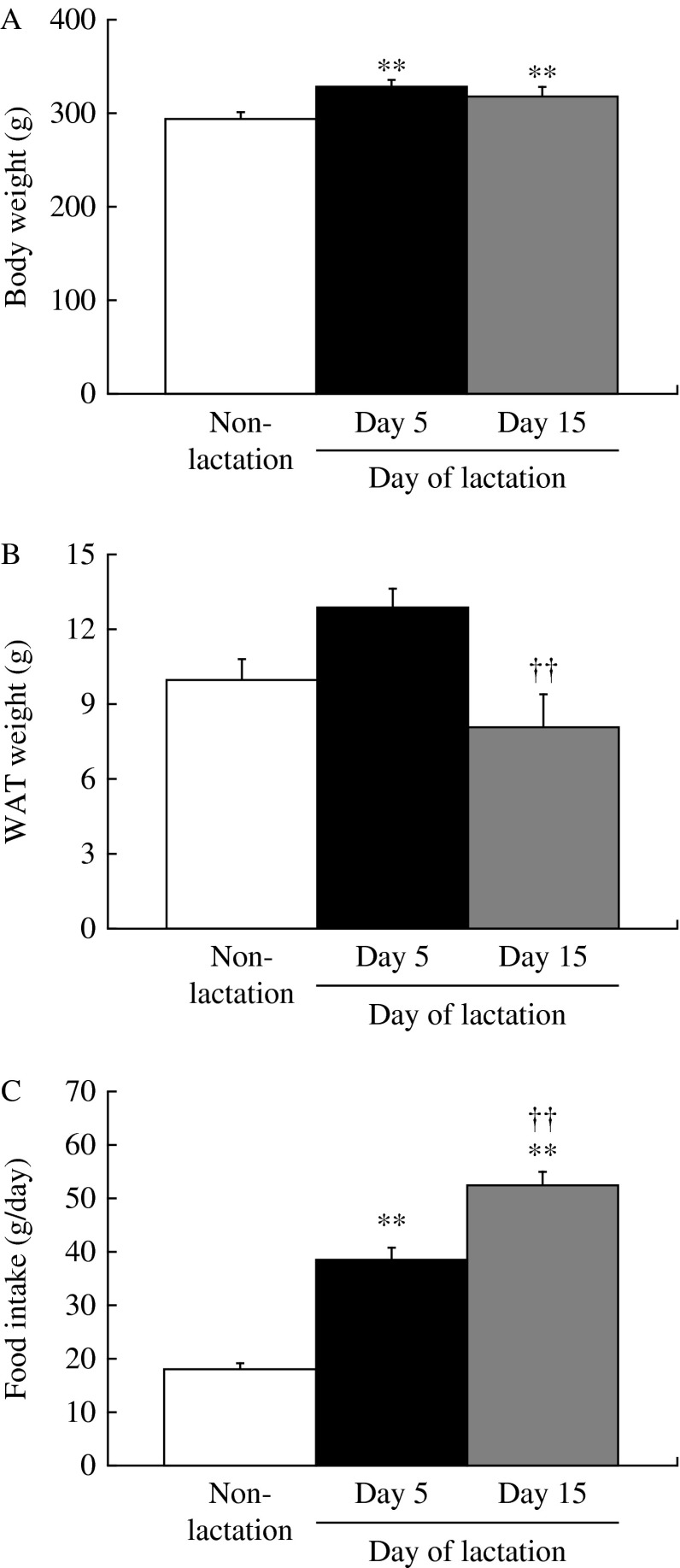
(A) Body weight, (B) WAT weight, and (C) food intake during 24 h in non-lactating rats (clear bars), and on day 5 (solid bars) and day 15 (gray bars) of lactation. Values are the means and vertical lines represent the s.e.m. (*n*=6). *P<0.05 vs non-lactation, **P<0.01 vs non-lactation, and ^††^*P*<0.01 vs day 5 of lactation.

**Figure 2:**
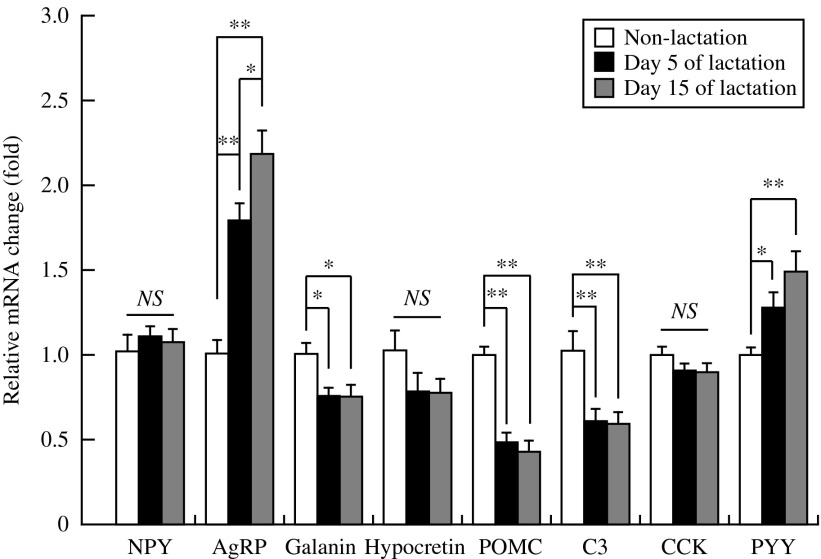
Relative changes in mRNAs for various hypothalamic appetite-regulating neuropeptides in non-lactating rats (clear bars), and on day 5 (solid bars) and day 15 (gray bars) of lactation. The mRNA level in non-lactating rats was assigned a value of 1. Values are the means and vertical lines represent the s.e.m. (*n*=6). *P<0.05 and **P<0.01.

**Figure 3:**
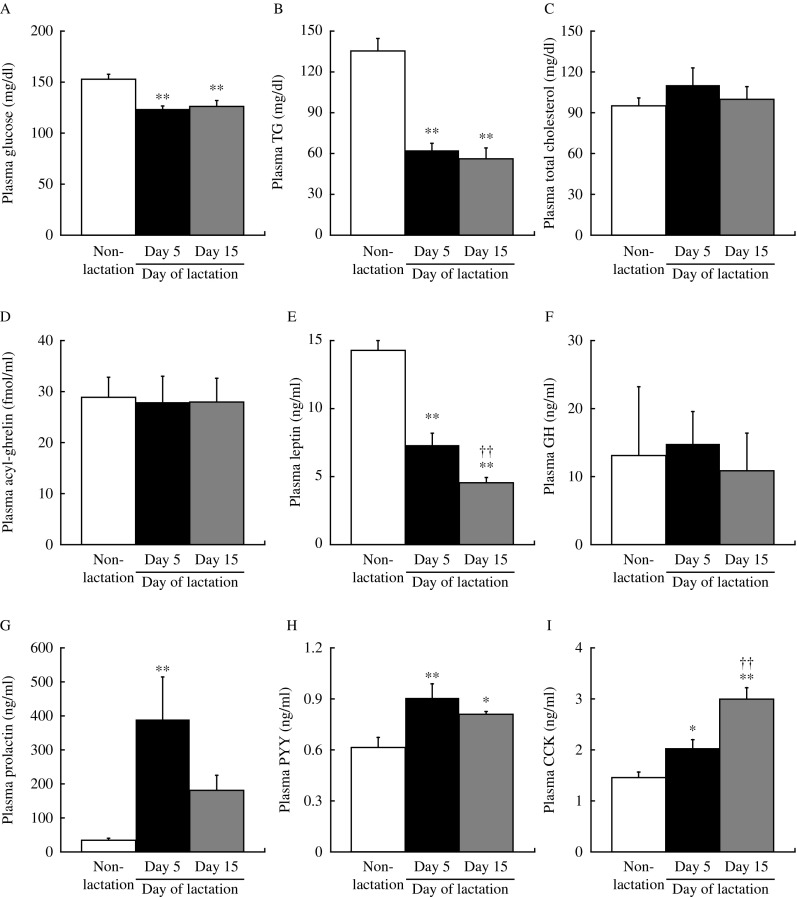
(A) Plasma glucose, (B) plasma triglyceride, (C) plasma total cholesterol, (D) plasma acyl-ghrelin, (E) plasma leptin, (F) plasma growth hormone, (G) plasma prolactin, (H) plasma PYY, and (I) plasma CCK levels in non-lactating rats (clear bars), and on day 5 (solid bars) and day 15 (gray bars) of lactation. Values are the means and vertical lines represent the s.e.m. (A, B, C, D, E and G, *n*=6; F: non-lactation: *n*=4, days 5 and 15 of lactation: *n*=6). **P<0.01 vs non-lactation and ^††^*P*<0.01 vs day 5 of lactation.

Body weight on days 5 and 15 of lactation was significantly (*P*<0.05) higher than in non-lactating female rats (Fig. 1A). Figure 1B shows the abdominal WAT weight in lactating (days 5 and 15) and non-lactating female rats. There were no significant differences in abdominal WAT weights between non-lactating rats and either of the lactation groups. Food intake on days 5 and 15 of lactation was significantly (*P*<0.01) higher than in non-lactating female rats (Fig. 1C). Food intake and abdominal WAT weight on day 15 of lactation were significantly (*P*<0.01) different than that on day 5. Table 1 shows the relative changes in mRNA expression for 84 kinds of hypothalamic appetite-regulating substances in non-lactating rats, and on days 5 and 15 of lactation by PCR array. During lactation, hypothalamic mRNA expression was significantly (*P*<0.05 and *P*<0.01) different from that in non-lactating female rats for the following: adenylate cyclase-activating polypeptide 1 receptor 1, adrenergic β-1 receptor, *Agrp*, *attractin*, *C3*, CART prepropeptide (*Cartpt*), *Cck*, ciliary neurotrophic factor receptor, dopamine receptor D1A (*Drd1a*), galanin prepropeptide, galanin receptor 1, GH receptor, ghrelin, hypocretin, hypocretin receptor type 1, insulin receptor, nuclear receptor subfamily 3 group C member 1, neurotrophic tyrosine kinase receptor type 1, *Pomc*, and *Pyy*. Figure 2 shows the relative mRNA expression for hypothalamic appetite-regulating neuropeptides in lactating (days 5 and 15) and non-lactating female rats. There were no significant differences in the mRNA expression for hypothalamic *Npy*, *Cck*, and hypocretin between non-lactating rats and both lactation groups. The hypothalamic *Agrp* and *Pyy* mRNA expression was significantly (*P*<0.05 and *P*<0.01) increased in both of the lactation groups relative to the non-lactation group. Lactating rats showed significantly (*P*<0.05) higher hypothalamic *Agrp* mRNA expression on day 15 than on day 5, but there was no significant difference in *Pyy* mRNA expression between days 5 and 15. Both lactation groups showed significantly (*P*<0.05 and *P*<0.01) lower hypothalamic *Pomc*, *C3*, and galanin mRNA expression than non-lactating rats. Figure 3 shows the plasma components in non-lactating rats, and on days 5 and 15 of lactation. On both days 5 and 15 of lactation, plasma glucose and TG levels were significantly (*P*<0.01) lower than in non-lactating rats (Fig. 3A and B). There were no significant differences in the levels of plasma total cholesterol, GH, and acyl-ghrelin between non-lactating rats and both lactation groups (Fig. 3C, D and F). The plasma leptin level was significantly (*P*<0.01) lower in each of the lactation groups than in non-lactating rats (Fig. 3E), but in lactating rats it was significantly lower (*P*<0.01) on day 15 than that on day 5. The plasma prolactin level was significantly (*P*<0.01) higher on day 5 of lactation than in non-lactating rats (Fig. 3G). The plasma PYY level was significantly (*P*<0.05 and *P*<0.01) higher on days 5 and 15 of lactation than in non-lactating rats (Fig. 3H). The plasma CCK level was significantly (*P*<0.05 and *P*<0.01) higher in each of the lactation groups (days 5 and 15) than in non-lactating rats, but in lactating rats that on day 15 was significantly (*P*<0.05) higher than on day 5 (Fig. 3I).

### Experiment 2

**Figure 4:**
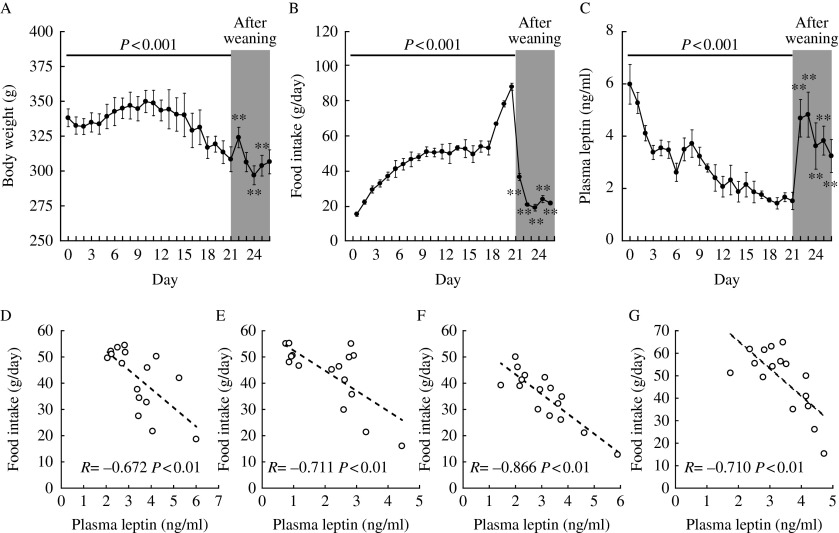
(A) Daily body weight, (B) daily food intake during 24 h, and (C) daily plasma leptin levels during lactation (days 0–21) and after weaning (days 22–26). Values are the means and vertical lines represent the s.e.m. (*n*=4). Correlations of food intake with the plasma leptin level during lactation (days 1–16) in individual dams (D, E, F and G, each 16 points). *P*<0.001 (ANOVA) during lactation. (A and C) **P<0.01 vs day 21 of lactation. (B) **P<0.01 vs day 16 of lactation.

Figure 4 shows the chronological changes in body weight, food intake, and plasma leptin level in female rats during lactation and after weaning, and the correlations of food intake with the plasma leptin level during days 1–16 of lactation for individual rats. Body weights changed significantly (*P*<0.001) during lactation. In comparison with body weight on day 21 of lactation, there was a significant (*P*<0.01) change on days 22, 24, and 25 (days 1, 3, and 4 after weaning) (Fig. 4A). Food intake was significantly (*P*<0.001) increased during the lactation period. Food intake after weaning was significantly (*P*<0.01) lower than on day 16 of lactation (Fig. 4B). Plasma leptin levels were significantly (*P*<0.001) decreased during lactation, but after weaning they were significantly (*P*<0.01) higher than on day 21 of lactation (Fig. 4C). During lactation (days 1–16), the plasma leptin level was negatively correlated with food intake in individual rats (*r*=−0.672 to −0.866, *P*<0.01; Fig. 4D, E, F and G).

### Experiment 3

**Figure 5:**
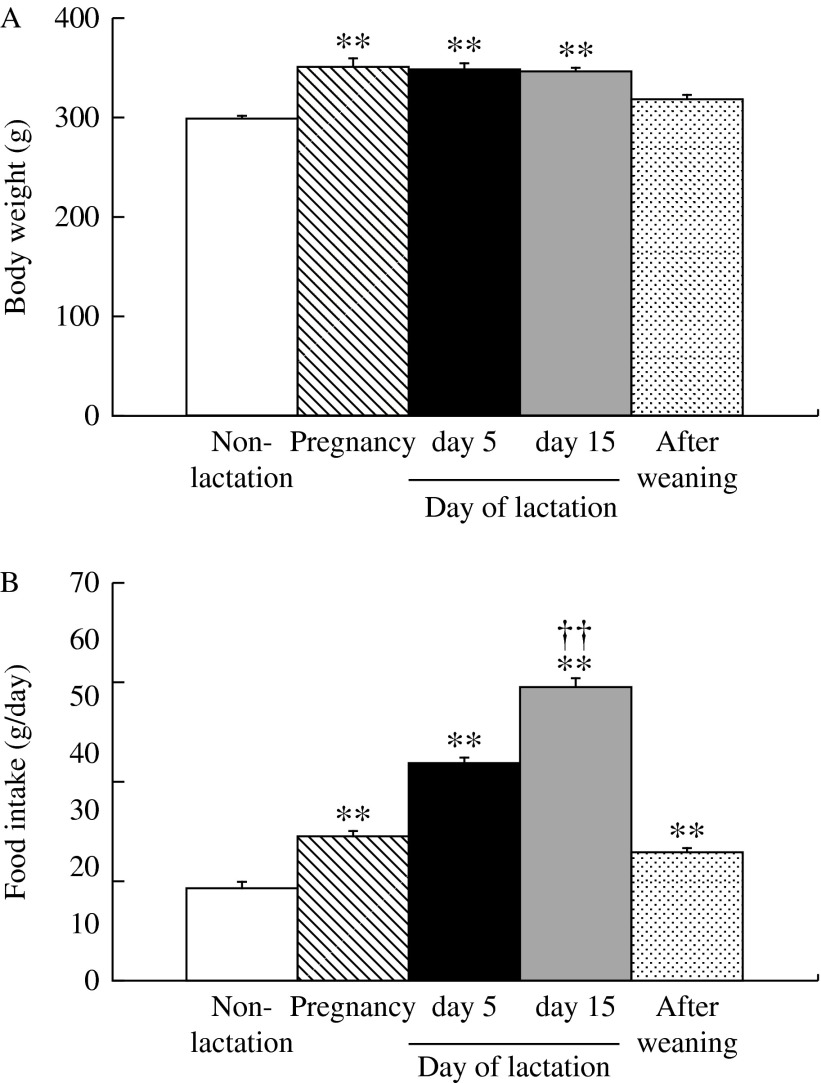
(A) Body weight and (B) food intake during 24 h in non-lactating rats (clear bars), and on day 14 of pregnancy (diagonal bars), day 5 (solid bars) and day 15 (gray bars) of lactation, and day 9 after weaning (dot bars). Values are the means and vertical lines represent the s.e.m. (*n*=6). **P<0.01 vs non-lactation, and ^††^*P*<0.01 vs day 5 of lactation.

**Figure 6:**
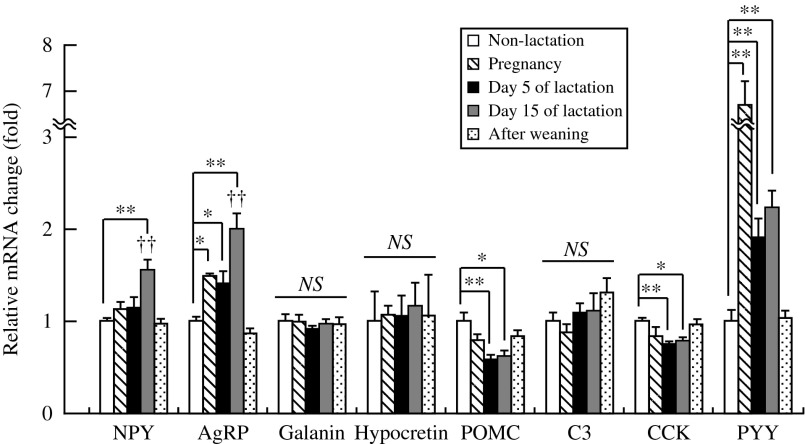
Relative changes in mRNAs for various arcuate nucleus appetite-regulating neuropeptides in non-lactating rats (clear bars), and on day 14 of pregnancy (diagonal bars), day 5 (solid bars) and day 15 (gray bars) of lactation, and day 9 after weaning (dot bars). The mRNA level in non-lactating rats was assigned a value of 1. Values are the means and vertical lines represent the s.e.m. (*n*=6). ^††^*P*<0.01 vs day 5 of lactation, *P<0.05, and **P<0.01.

Body weight and food intake during pregnancy, and on days 5 and 15 of lactation were significantly (*P*<0.01) higher than in non-lactating female rats (Fig. 5A and B). Food intake on after weaning was significantly (*P*<0.01) higher than in non-lactating female rats. Food intake on day 15 of lactation was significantly (*P*<0.01) different from that on day 5. Figure 6 shows the relative mRNA expression for ARC appetite-regulating neuropeptides at all stages of pregnancy, lactation (days 5 and 15), and after weaning, and in non-lactating female rats. During pregnancy and lactation (days 5 and 15), *Agrp* and *Pyy* mRNA expression was significantly (*P*<0.05 and *P*<0.01) higher than in non-lactating rats. On day 15 of lactation, rats showed significantly (*P*<0.01) higher *Npy* mRNA expression than non-lactating rats. Both lactation groups (days 5 and 15) showed significantly (*P*<0.01) lower *Pomc* and *Cck* mRNA expression than non-lactating rats. There were no significant differences in the mRNA expression for *C3*, galanin and hypocretin between non-lactating rats and other groups. Lactating rats showed significantly (*P*<0.05) higher *Agrp* and *Npy* mRNA expression on day 15 than on day 5, but there was no significant difference in the *Pyy* mRNA expression between days 5 and 15.

## Discussion

In order to clarify the mechanism responsible for induction of hyperphagia on lactation in female rats, we analyzed the daily food intake, plasma leptin levels, and mRNA expression of appetite-regulating peptides in ARC. Results demonstrating mRNA expression levels of appetite-regulating peptides in ARC of lactating rats suggested that *Agrp*, *Npy* (only on day 15 of lactation), and *Pyy* were upregulated, whereas *Pomc*, *Cck* were downregulated, in comparison with non-lactating rats. Food consumption was two to three times higher in lactating rats, and increased with the course of lactation. Crowley *et al*. (2003) reported that chronic administration of an NPY antagonist into the third ventricle had a less marked effect on feeding in lactating rats than in non-lactating rats. This suggested that the continued hyperphagia accompanying the course of lactation might be closely associated with increased hypothalamic *Agrp* mRNA expression. On the other hand, it has been reported that lactating rats show significantly lower mRNA expression for the hypothalamic anorexigenic peptide POMC than non-lactating rats (Smith 1993, Mann *et al*. 1997). The activity of POMC neurons is inhibited by the activity of AgRP neurons (Cone 2005, Tong *et al*. 2008). Furthermore, a population of neurons in ARC is in direct contact with the circulation and shows higher sensitivity to circulating leptin than neurons behind the BBB in the hypothalamus (Faouzi *et al*. 2007). This study showed that the plasma leptin level decreased continually during the continued hyperphagia accompanying the course of lactation. In this study, the increased *Agrp* mRNA expression in ARC may have contributed to the decreased *Pomc* mRNA expression in ARC and circulating leptin levels during lactation.

Food consumption was significantly higher on days 5 and 15 of lactation than in non-lactating rats. PYY, a member of the NPY peptide family secreted primarily from endocrine L cells, binds to the Y2 NPY receptor and inhibits the activation of NPY/AgRP neurons (Moran 2006, Schwartz 2006). In addition, both hypothalamic immunoreactivity and *Pyy* mRNA expression have been observed in rat and human (Ekman *et al*. 1986, Morimoto *et al*. 2008). Batterham *et al*. (2002) reported that food intake in male rats was reduced after injection of PYY into ARC. However, our present results suggested that mRNA expression for anorexigenic PYY in ARC was higher in pregnant and lactating rats than in non-lactating rats. Aguilar *et al*. (2004) reported that i.c.v. administration of PYY in hyperprolactinemic female rats promoted secretion of prolactin in the anterior pituitary. Moreover, their group showed that PYY directly stimulated secretion of luteinizing hormone and follicle-stimulating hormone at the pituitary level in prepubertal female rats (Fernandez-Fernandez *et al*. 2005). Therefore, in pregnant and lactating female rats, the increased *Pyy* mRNA expression in ARC may promote the secretion of gonadotropin and prolactin. Further studies will be required to investigate the central effects of PYY during pregnancy and lactation. Hypocretin, an orexigenic hormone, binds to the orexin1 and orexin2 receptors and activates NPY neurons (Sakurai *et al*. 1998, Dube *et al*. 1999, Yamanaka *et al*. 2000). There was no difference in the mRNA expression for orexigenic hypocretin in ARC between non-lactating rats and both pregnant and lactating rats, and also post-weaning rats. Hypocretin may thus not play a role in the stimulation of food intake during lactation, as has been reported previously (Brogan *et al*. 2000, Sun *et al*. 2003). CCK, an anorexigenic molecule secreted primarily from intestinal I-cells, binds to the CCK1 receptor and activates POMC neurons in the NTS via the vagus nerve (Fan *et al*. 2004, Millington 2007). In addition, hypothalamic *CCK* mRNA expression has been observed in rats and humans (Iadarola *et al*. 1989, Ding & Bayer 1993). During lactation, although *CCK* mRNA expression in ARC was lower than that in non-lactating female rats, the level of *CCK* in plasma was increased. Chen *et al*. (2008) reported that administration of CCK in the dorsomedial hypothalamus (DMH) decreased food intake and the level of *NPY* mRNA in DMH, and increased the number of c-Fos positive cells in ARC. In addition, their results suggested that peripheral administration of CCK did not increase the number of c-Fos positive cells in ARC. Therefore, lower expression of *CCK* mRNA in ARC may be involved in the long-term hyperphagia characteristic of lactation.

In this study, the hypothalamic *C3* mRNA expression in lactating rats was lower than that in non-lactating rats. However, there were no differences in *C3* mRNA expression in ARC between non-lactating rats and other groups. C3, a complement protein, binds to the C3a receptor (C3aR) after it has been cleaved to C3a and C3b. C3 is cleaved to C3a and C3b by C3 convertase (Koch *et al*. 2012). Therefore it is suggested that the level of C3a in the brain of lactating rats is lower than in non-lactating rats. Ohinata *et al*. (2002) reported that, in male mice, intraventricular administration of C3a reduced food intake, and that C3a receptor activity suppressed food intake through production of PGE2 and EP4 receptor activity (Ohinata *et al*. 2007). Therefore, the decreased hypothalamic *C3* mRNA expression (except for ARC) in lactating rats may be indirectly involved in the hyperphagia associated with lactation. Further research will be required to investigate the levels of C3 convertase and the suppressive effects of C3a on appetite during lactation.

Our results also suggested that plasma prolactin levels were increased in lactating dams. During lactation, prolactin and oxytocin play important roles in milk production and ejection. Galanin, an orexigenic hormone secreted from the gut, binds to its receptors GalR1, GalR2, and GalR3. In ARC and the paraventricular nucleus, galanin activates NPY neurons by binding to GalR1 (Fang *et al*. 2012). Furthermore, oxytocin and galanin neurons have been identified in the hypothalamic supraoptic nucleus (Melnikova *et al*. 2006, Sakamoto *et al*. 2008). Galanin suppresses the hypothalamic oxytocin mRNA expression (Ciosek & Cisowska 2003). Although one of the effects of galanin is to increase food intake, we confirmed that the hypothalamic galanin mRNA expression was lower in lactating, than in non-lactating, rats as has been reported previously (Landry *et al*. 1997). In addition, there were no differences in galanin mRNA expression in ARC between non-lactating rats and other groups. Thus, the decreased hypothalamic galanin mRNA expression in lactating rats may not suppress release of oxytocin from the hypothalamus for milk ejection.

With regard to changes in blood components, the plasma levels of TG and glucose were decreased on days 5 and 15 of lactation, relative to those in non-lactating rats. These results are consistent with previously reported data for lactating rats (Jones *et al*. 1984, Martínez *et al*. 2002). Blood glucose and TG in lactating rats are important resources for mammary gland milk production, and their decline may reflect their depletion during milk production. It has been reported that the circulating levels of PYY and CCK increase postprandially (Coll *et al*. 2007, Zwirska-Korczala *et al*. 2007). The circulating levels of PYY and CCK in lactating rats were also higher than in non-lactating rats, as has been reported previously (Lindén *et al*. 1990, Taylor *et al*. 2009). However, *Npy* mRNA expression in ARC on day 15 of lactation was higher than in non-lactating female rats. Peripheral PYY and CCK signals arrive at NTS via the vagal afferent pathway (Fan *et al*. 2004, Koda *et al*. 2005, Millington 2007). Ladyman *et al*. (2011) reported that i.p. injection of CCK in pregnant rats did not significantly decrease food intake or increase the number of c-Fos positive cells in the hypothalamic paraventricular nucleus and supraoptic nucleus. Therefore, during lactation, peripheral PYY and CCK may be reduced sensitivity via the vagal afferent pathway.

This study showed that the plasma leptin level decreased continually during the course of lactation. In comparison with non-lactating rats, WAT weight during lactation was either unchanged (day 15) or increased (day 5). However, the plasma leptin level during lactation was lower than in non-lactating rats. In addition, on day 1 after weaning, the leptin level was suddenly increased relative to the day before weaning (day 21 of lactation). Synthesis of leptin is promoted by insulin stimulation in WAT (Barr *et al*. 1997). It has also been reported that prolactin is involved in insulin resistance in WAT during lactation (Graham *et al*. 1990) and suppresses insulin-induced leptin production in WAT (Ling & Billig 2001). In this study, plasma prolactin levels in lactating rats were significantly higher than in non-lactating rats. It has been reported that after weaning, the plasma level of prolactin declines in non-lactating female rats within a few hours (van Landehem & van de Wiel 1978). In addition, Brogan *et al*. (1999) reported that initiation of milk production, but not suckling stimulus alone, participated in the decrease in the serum leptin level during lactation. Therefore, the decrease in the plasma leptin level during lactation may reflect not only the decrease of WAT weight but also the increase in the prolactin level in response to the suckling stimulus. Furthermore, cancellation of the suppression of leptin secretion by stopping milk production may be involved in the decreased food intake after weaning. Leptin suppresses the activity of NPY/AgRP neurons and increases that of POMC neurons in ARC (Schwartz *et al*. 2000). The daily plasma leptin level during lactation (days 1–16) was negatively correlated with food intake. In addition, our previous study demonstrated that the hyperphagia of lactating rats could be partly due to reduced sensitivity of neurons to leptin in blood (Suzuki *et al*. 2010). These results suggest that increased *Agrp* mRNA expression and decreased *Pomc* mRNA in ARC expression in lactating rats contribute to a decline in both leptin secretion and sensitivity.

Acyl-ghrelin, an orexigenic hormone secreted from the stomach, binds to the GH secretagogue receptor (GHS-R) and stimulates the release of NPY in the hypothalamus (Nakazato *et al*. 2001, Wren *et al*. 2001). Although food consumption increased during the course of lactation, many studies have demonstrated no change in, or reduced, levels of acyl-ghrelin in the blood of lactating rats (Abizaid *et al*. 2008, Taylor *et al*. 2009). In this study, we confirmed that the plasma acyl-ghrelin levels also did not differ between non-lactating rats and both of the lactation groups. Abizaid *et al*. (2008) reported that rats on day 15 of lactation showed increased hypothalamic acyl-ghrelin receptor (GHS-R1a) mRNA expression relative to non-lactating rats, and that the expression returned to the baseline after litter removal. In addition, milk production in lactating rats is reportedly increased by chronic administration of acyl-ghrelin (Nakahara *et al*. 2003). Therefore, acyl-ghrelin in lactating rats may play an important role in milk production through suckling stimulation rather than through regulation of appetite.

In conclusion, this study has clarified the synchronous changes in the levels of many appetite-regulating peptides during pregnancy, lactation, and after weaning in female rats. Our findings suggest that the hyperphagia associated with lactation is caused by decreased *Pomc* and *Cck* and increased *Agrp* mRNA expression in ARC. A noteworthy finding was that *Agrp* mRNA expression in ARC was associated with a decrease in the blood leptin level during course of lactation. In lactating rats, therefore, *Agrp* mRNA expression in ARC and the level of leptin in blood may be involved in the continued hyperphagia accompanying the course of lactation.

## Supplementary data

This is linked to the online version of the paper at http://dx.doi.org/10.1530/JME-13-0015.

Supplemental Figure

## Figures and Tables

**Table 1:** Relative changes in mRNAs for 84 kinds of hypothalamic appetite-regulating substances in non-lactating rats, and on days 5 and 15 of lactation, determined by PCR array. The mRNA level in non-lactating rats was assigned a value of 1. Values are the means and the s.e.m. (*n*=4)

**Gene names**	**Fold change per non-lactation**		**ANOVA** (*P* value)
Day 5 of lactation	Day 15 of lactation
Adenylate cyclase activating polypeptide 1	1.06±0.04	1.05±0.09	–
Adenylate cyclase activating polypeptide 1 receptor 1	0.56±0.03	0.51±0.01	†
Adiponectin, C1Q and collagen domain containing	2.13±1.08	0.47±0.05	–
Adiponectin receptor 1	0.91±0.02	0.95±0.03	–
Adiponectin receptor 2	0.89±0.08	1.09±0.05	–
Adrenergic α-2B receptor	0.92±0.15	0.97±0.10	–
Adrenergic β-1 receptor	0.69±0.03	0.61±0.07	†
Agouti-related protein homolog	1.74±0.14	2.13±0.27	†
Apolipoprotein A–IV	0.42±0.21	0.44±0.26	–
Attractin	0.84±0.04	0.85±0.04	*
Brain-derived neurotrophic factor	1.08±0.04	1.20±0.08	–
Bombesin-like receptor 3	0.98±0.12	1.29±0.20	–
Complement component 3	0.51±0.11	0.52±0.05	*
Calcitonin-related polypeptide α	1.01±0.23	0.93±0.19	–
Calcitonin receptor	0.97±0.06	0.93±0.05	–
CART prepropeptide	0.78±0.05	0.85±0.07	*
Cholecystokinin	0.74±0.02	0.71±0.03	*
Cholecystokinin A receptor	0.94±0.03	1.08±0.21	–
Colipase, pancreatic	1.17±0.11	1.17±0.27	–
Cannabinoid receptor 1	0.83±0.05	0.92±0.03	–
Ciliary neurotrophic factor	0.81±0.03	0.89±0.09	–
Ciliary neurotrophic factor receptor	0.70±0.03	0.87±0.06	*
Corticotropin-releasing hormone	1.00±0.08	0.85±0.11	–
Corticotropin-releasing hormone receptor 1	0.80±0.03	0.95±0.09	–
Dopamine receptor D1A	0.68±0.06	0.78±0.05	†
Dopamine receptor D2	0.83±0.06	0.97±0.08	–
Galanin prepropeptide	0.74±0.03	0.71±0.08	*
Galanin receptor 1	0.90±0.04	0.76±0.04	†
Glucagon	1.30±0.30	1.35±0.85	–
Glucagon receptor	1.30±0.88	3.20±0.79	–
Growth hormone 1 (GH1)	1.72±0.62	2.03±0.46	–
GH receptor	0.91±0.01	0.98±0.01	*
Ghrelin/obestatin prepropeptide	0.88±0.03	1.10±0.04	†
GH secretagogue receptor	0.84±0.01	0.95±0.11	–
Glucagon-like peptide 1 receptor	1.15±0.05	1.30±0.13	–
Prolactin-releasing hormone receptor	1.09±0.08	1.06±0.09	–
Melanin-concentrating hormone receptor 1	0.98±0.07	0.99±0.04	–
Gastrin-releasing peptide	0.93±0.06	1.08±0.11	–
Gastrin-releasing peptide receptor	0.87±0.13	0.86±0.15	–
Hypocretin	0.63±0.05	0.64±0.07	†
Hypocretin receptor 1	0.72±0.05	0.73±0.05	†
Histamine receptor H1	0.87±0.11	0.89±0.02	–
5-Hydroxytryptamine receptor 2C	1.07±0.12	1.11±0.07	–
Islet amyloid polypeptide	3.35±1.05	2.88±1.86	–
Interleukin 1α	0.79±0.20	0.94±0.24	–
Interleukin 1β	0.74±0.19	0.53±0.06	–
Interleukin 1 receptor, type 1	0.65±0.06	0.78±0.13	–
Interleukin 6	1.18±0.16	1.27±0.47	–
Interleukin 6 receptor	0.69±0.08	0.80±0.16	–
Insulin 1	1.73±0.28	0.98±0.32	–
Insulin 2	0.99±0.25	0.61±0.19	–
Insulin receptor	0.78±0.03	0.86±0.06	*
Leptin	1.28±0.74	1.35±0.57	–
Leptin receptor	0.90±0.04	1.20±0.15	–
Melanocortin 3 receptor	0.87±0.07	0.93±0.11	–
Neuromedin B	0.88±0.09	1.06±0.10	–
Neuromedin B receptor	0.94±0.07	1.00±0.14	–
Neuromedin U	1.12±0.39	0.60±0.27	–
Neuromedin U receptor 1	0.72±0.11	1.05±0.21	–
Neuropeptide Y	1.00±0.04	1.06±0.09	–
Neuropeptide Y receptor Y1	0.89±0.07	0.91±0.06	–
Nuclear receptor subfamily 3, group C, member 1	0.75±0.05	0.90±0.04	†
Neurotrophic tyrosine kinase, receptor, type 1	0.39±0.08	0.30±0.03	†
Neurotensin	1.13±0.12	1.00±0.17	–
Neurotensin receptor 1	0.91±0.10	0.98±0.09	–
Opioid receptor, κ1	0.88±0.06	1.04±0.04	–
Opioid receptor, μ1	1.00±0.06	0.97±0.04	–
Sigma non-opioid intracellular receptor 1	0.96±0.06	1.00±0.02	–
Proopiomelanocortin	0.47±0.04	0.34±0.05	†
Peroxisome proliferator-activated receptor α	0.82±0.03	0.99±0.09	–
Peroxisome proliferator-activated receptor γ	0.93±0.25	0.83±0.06	–
Peroxisome proliferator-activated receptor γ, coactivator 1α	0.84±0.03	0.98±0.02	–
Protein tyrosine phosphatase, non-receptor type 1	0.76±0.02	0.82±0.09	–
Peptide YY	2.15±0.22	2.37±0.49	*
Receptor (G protein-coupled) activity modifying protein 3	0.90±0.05	0.90±0.10	–
Sortilin 1	0.87±0.03	1.01±0.09	–
Somatostatin	0.87±0.09	0.85±0.01	–
Somatostatin receptor 1	0.82±0.08	0.77±0.08	–
Thyroid hormone receptor β	0.89±0.04	0.90±0.07	–
Tumor necrosis factor	1.06±1.06	1.01±0.21	–
Thyrotropin-releasing hormone	0.94±0.94	0.89±0.04	–
Thyrotropin-releasing hormone receptor	0.97±0.97	0.97±0.08	–
Urocortin	1.12±1.12	1.21±0.41	–
Uncoupling protein 1	1.33±1.33	1.50±0.25	–

*P<0.05 (ANOVA) and ^†^*P*<0.01 (ANOVA).
