# Supplementary material for: Changes in mRNA expression of arcuate nucleus appetite-regulating peptides during lactation in rats
Source: J Mol Endocrinol. 2013 Dec 3;52(2):97–109. doi: 10.1530/JME-13-0015 (PMC3907180; doi:10.1530/JME-13-0015)
Supplement: Supplemental Figure [file supp_52_2_97__index.html]

Changes in mRNA expression of arcuate nucleus appetite-regulating peptides during lactation in rats — Lactation changes appetite-regulating peptides — Supplemental Figure 

# Changes in mRNA expression of arcuate nucleus appetite-regulating peptides during lactation in rats

## Supplementary Figure

**Files in this Data Supplement:**

- Supplementary Figure 1 - The diagram of each experiment timeline. (PDF 83 KB)
