# Supplementary figures and images for: Changes in mRNA expression of arcuate nucleus appetite-regulating peptides during lactation in rats
Source: J Mol Endocrinol. 2013 Dec 3;52(2):97–109. doi: 10.1530/JME-13-0015 (PMC3907180; doi:10.1530/JME-13-0015)

**Experiment.1 (Hypothalamus sampling)**

**↓ = Sampling**

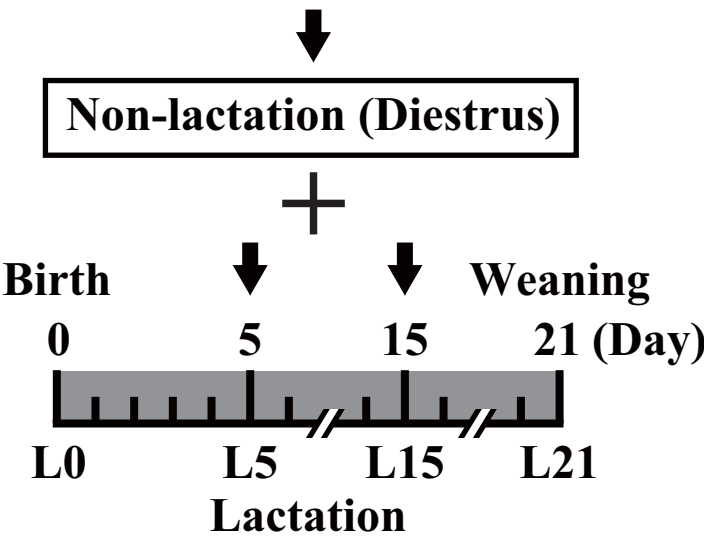

**Experiment.2**

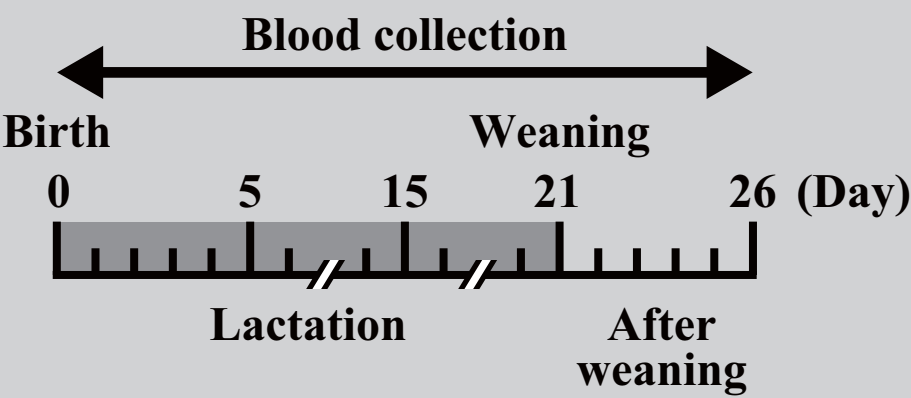

**Experiment.3 (ARC sampling)**

**↓ = Sampling**

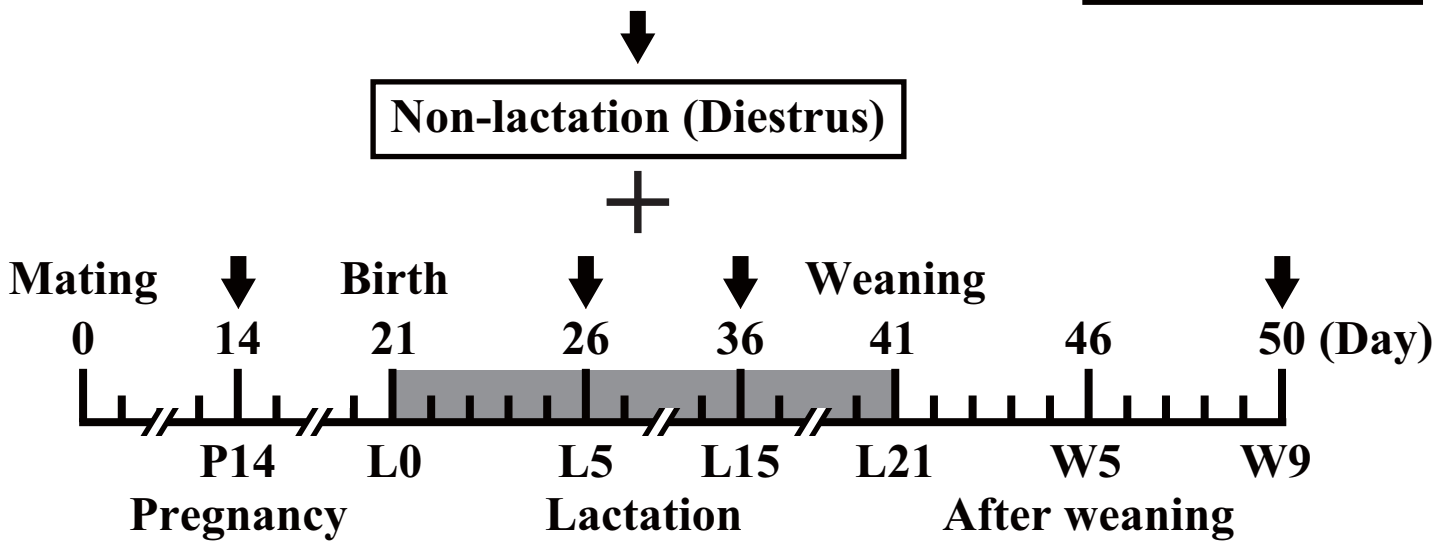

Supplement: Supplemental Figure [file supp_JME-13-0015_Supplementary_figure_1.pdf]
